# Supplementary material for: Local BMP2 hydrogel therapy for robust bone regeneration in a porcine model of Legg-Calvé-Perthes disease
Source: NPJ Regen Med. 2023 Sep 14;8:50. doi: 10.1038/s41536-023-00322-2 (PMC10502123; doi:10.1038/s41536-023-00322-2)
Supplement: Supplementary file 1 — supplemental material [file 41536_2023_322_MOESM1_ESM.pdf]

Supplementary information

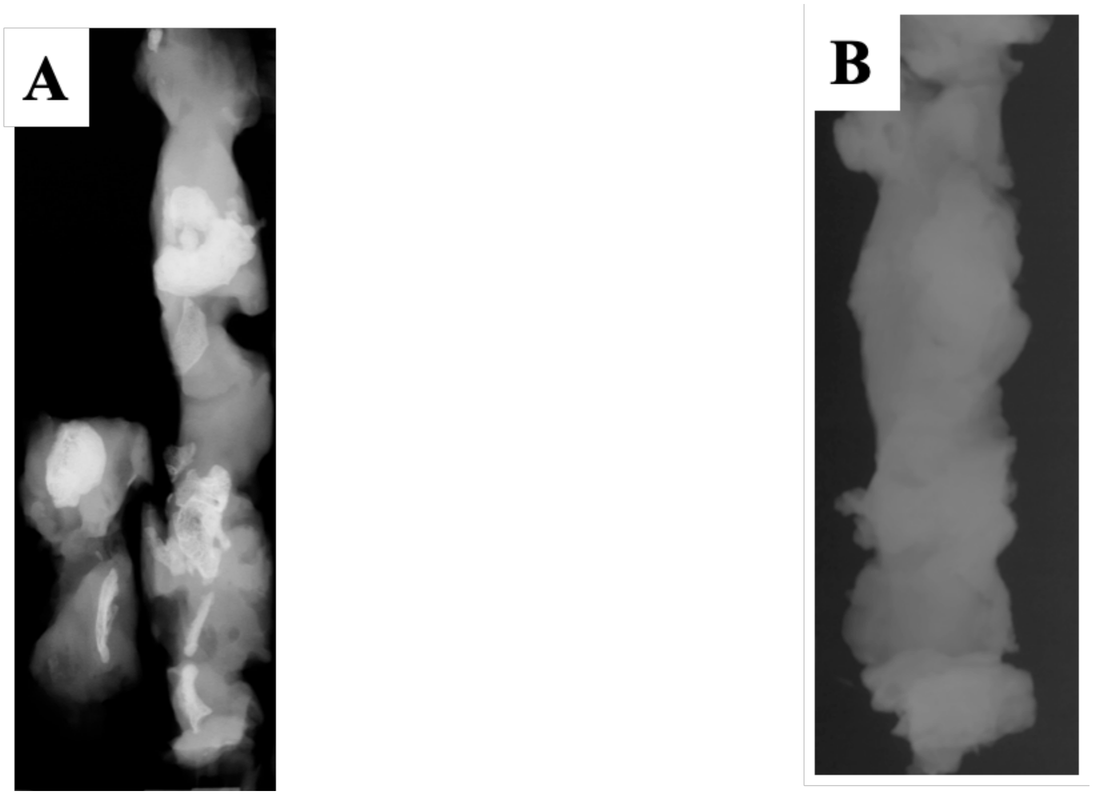

Supplementary Figure 1. X-ray images showing the dissected hip joint capsules and the soft tissues. A) The BMP2 saline treatment showing the obvious HO formation. (*21*) B) The BMP2 hydrogel treatment showing no HO formation.

Supplementary Table 1: Quantitative RT-PCR primers used in the study.

Gene name	Forward primer	Reverse primer
Col1	5'- GAGGCCGTTCTGTACGCAG-3'	5'- GATTGAGGGACCTGGAGCCC-3'
ALP	5'- AAGACACACTGACCGTCGTC-3'	5'- GGGGGCCAGACCAAAGATAG-3'
Osx	5'- CTCATTCCCTGGCTCAC-3'	5'- TGGGCAGACAGTCAGAAGAG-3'
b-Actin	5'- CACGCCATCCTGCGTCTGGA-3'	5'- AGCACCGTGTTGGCGTAGAG-3'
